# Rapid flow cytometric measurement of protein inclusions and nuclear trafficking

**DOI:** 10.1038/srep31138

**Published:** 2016-08-12

**Authors:** D. R. Whiten, R. San Gil, L. McAlary, J. J. Yerbury, H. Ecroyd, M. R. Wilson

**Affiliations:** 1 Illawarra Health and Medical Research Institute and School of Biological Sciences, University of Wollongong, Wollongong NSW2522, Australia.

## Abstract

Proteinaceous cytoplasmic inclusions are an indicator of dysfunction in normal cellular proteostasis and a hallmark of many neurodegenerative diseases. We describe a simple and rapid new flow cytometry-based method to enumerate, characterise and, if desired, physically recover protein inclusions from cells. This technique can analyse and resolve a broad variety of inclusions differing in both size and protein composition, making it applicable to essentially any model of intracellular protein aggregation. The method also allows rapid quantification of the nuclear trafficking of fluorescently labelled molecules.

A pathological hallmark of neurodegenerative diseases such as amyotrophic lateral sclerosis (ALS) and Huntington’s disease is the *de novo* misfolding of endogenous proteins, which oligomerize and aggregate to exert cytotoxicity^1^,^2^,^3^. In an attempt to reduce toxicity and prevent the prion-like propagation of misfolded protein, protein quality control mechanisms, collectively called the proteostasis network, may sequester these aggregation-prone proteins into inclusion bodies^2^,^4^,^5^,^6^. Despite this strategy, there is a strong correlation between inclusion formation and subsequent neuronal death^7^. A well established technique to study the formation of insoluble protein inclusions within mammalian cells is to over-express one or more aggregation-prone proteins fused to a fluorescent protein^8^,^9^.

Until recently, methods available to researchers to quantify inclusions such as these in cells were largely limited to crude centrifugation methods, “filter trap assays” (where cells were lysed in detergents and the insoluble inclusions trapped on a filter membrane), and the often laborious manual counting of inclusions in images captured by microscopy. Fluorescence microscopy enables the visualization and quantification of fluorescently-tagged protein aggregates in cells but is severely limited in terms of its throughput and quantitation. Despite recent advances in automated imaging^10^,^11^, this approach still suffers from significant limitations, including inadequate discrimination between individual cells or inclusions that occur in clusters, and compromised performance for images with high background fluorescence. It should also be noted that the previously available methods are variably well suited to the quantification of inclusions formed by different proteins, meaning that in the past it was problematic to compare results between different protein aggregation models or between studies.

To answer the need for a versatile, high-throughput method to detect a diversity of protein inclusions in cells, we developed FloIT (flow cytometric analysis of inclusions and trafficking) - a sensitive and robust flow cytometric technique in which the number of inclusions present in a cell lysate is normalised against the number of nuclei to allow quantitative comparisons between treatments (in this way transfection efficiencies can also be taken into account). We also show that the same technique can be used to measure the trafficking of fluorescent molecules into and out of the nucleus.

## Results and Discussion

**Figure 1:**
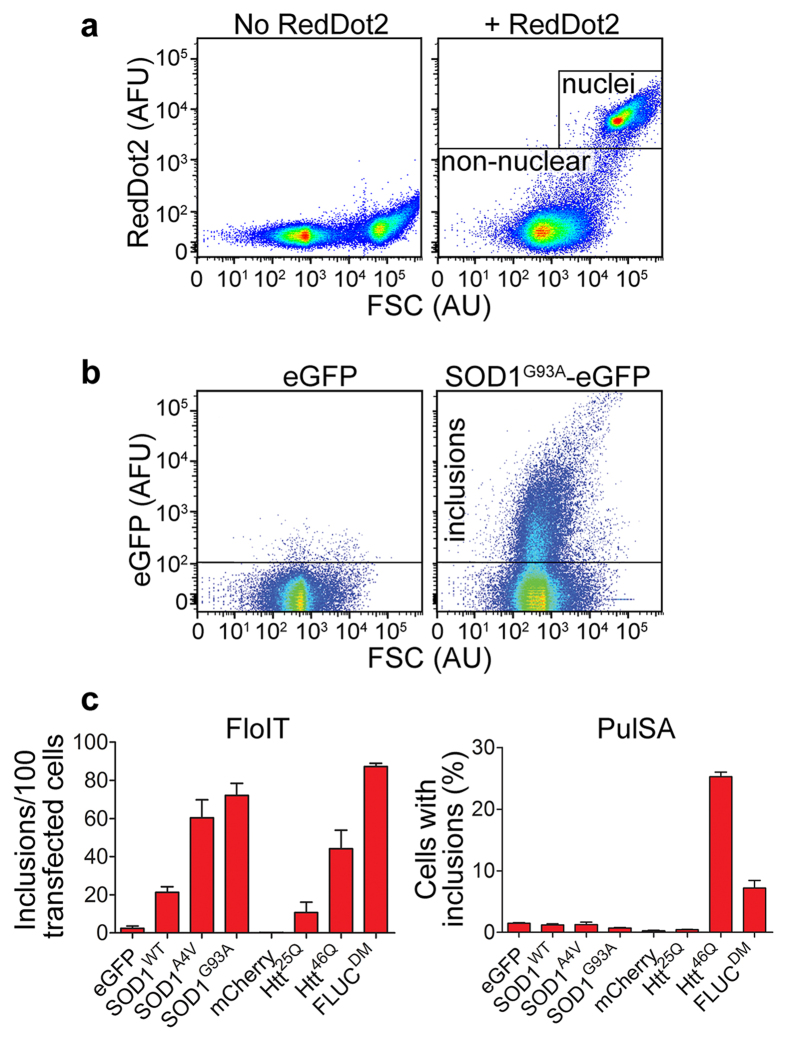
FloIT detects inclusions formed by a wide variety of proteins. (**a**) Two parameter, pseudo-colour flow cytometry plots showing identification of nuclei and non-nuclear particles (indicated) using FSC and RedDot2 fluorescence (*left:* unstained, *right:* stained with RedDot2). (**b**) Non-nuclear particles (gated as shown in (**a**)) analysed for eGFP fluorescence versus FSC in lysates prepared from cells transfected to express eGFP (*left*) or SOD1^G93A^-eGFP (*right*). SOD1^G93A^-eGFP inclusions (indicated) are identified by their increased eGFP fluorescence. (**c**) Detection of inclusions (FloIT, *left*) and cells containing inclusions (PulSA, *right*) formed by various proteins. Data are means (n = 3) ± SEM (too small to be visible in some cases). All experiments used N2a cells and each result is representative of two or more independent experiments.

The evolution of FloIT was inspired to some extent by an earlier report that mechanically burst open yeast cells to quantify fluorescent protein aggregates by flow cytometry (normalised against the protein content of the lysate)^12^. To initially develop the FloIT technique, cultured Neuro-2a (N2a) cells were transfected (or not) to over-express G93A superoxide dismutase-1 fused at the C-terminus with enhanced green fluorescent protein (SOD1^G93A^-eGFP). The cells were subsequently lysed in 0.5% (v/v) Triton X-100 in phosphate buffered saline (PBS) and the nuclei stained with RedDot2 before flow cytometric analysis. Mammalian cell inclusions, which are comprised of extensively misfolded and aggregated proteins, are well known to be highly resistant to detergents^13^. Nuclei were identified (and quantified) using forward scatter (FSC) and RedDot2 fluorescence (Fig. 1a, *right*). By exploiting the eGFP tag on the aggregating SOD1^G93A^ protein, non-nuclear particles were then gated into inclusions and cellular debris (Fig. 1b, *right*). To calculate the average number of inclusions per transfected cell in the population analysed, the number of inclusions acquired is simply divided by the transfection efficiency multiplied by the corresponding number of cell nuclei enumerated (the fraction of cells transfected is separately quantified by flow cytometry).

By plotting FSC-Height versus FSC-Width for RedDot2-stained particles, it is possible to separately enumerate single nuclei, nuclear doublets, and larger nuclear aggregates in cell lysates (see Supplementary Fig. S1a). For N2a cell lysates, approximately 95% of the RedDot2-stained particles enumerated are single nuclei, with the remainder comprised of mostly nuclear doublets and much smaller numbers of larger nuclear aggregates (Supplementary Fig. S1a). The non-singlet nuclear particles enumerated thus incorporate about 10% of the total numbers of cell nuclei. This means that simply enumerating all RedDot2-stained particles in N2a cell lysates (as depicted in Fig. 1a) will underestimate the total number of single nuclei by about 5%, a small error that is constant between samples. The numbers of nuclear particles enumerated in N2a cell lysates (gated as shown in Fig. 1a) does not change significantly for at least 3 h under the conditions tested (Supplementary Fig. S1b), confirming limited aggregation of N2a nuclei under the conditions used. FloIT data presented in this report in units of inclusions/100 nuclei has been corrected for the 5% underestimation of the numbers of single nuclei resulting from gating as in Fig. 1a. Note, however, that many FloIT analyses are likely to focus on the effects of treatments on the relative (and not absolute) numbers of inclusions per cell, meaning that this small correction may not always be required.

**Figure 2:**
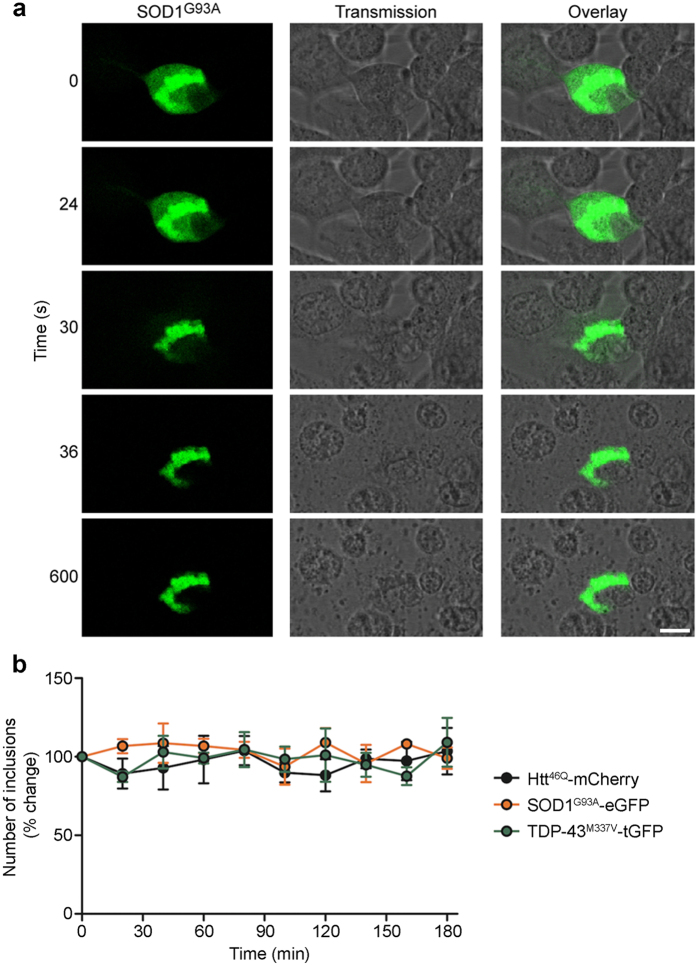
Protein inclusions remain insoluble in lysis buffer and FloIT analyses can be performed up to several hours post-lysis. (**a**) Time series of confocal microscopy images showing cells expressing SOD1^G93A^-eGFP imaged every 6 s following the addition of lysis buffer. The release of soluble SOD1^G93A^ from the newly lysed cell is visible at 30 s while the inclusions remained unchanged for at least 10 minutes. Scale bar (bottom right) is 5 μm. (**b**) N2a cells transfected to express the indicated protein were lysed at a density of 150,000 cells/ml. The lysates were kept on ice over the entire 3 h time course and were mixed briefly before aliquots were taken for FloIT measurement at the times indicated. Data are plotted as % of t = 0 values and are means ± SEM (n = 3). Results shown are each representative of two independent experiments.

To demonstrate that FloIT was broadly applicable, we extended this initial work to encompass a variety of additional protein aggregation models including SOD1^WT^-eGFP and SOD1^A4V^-eGFP, a conformationally destabilised double mutant of firefly luciferase fused to eGFP (R188Q, R261Q; FLUC^DM^-eGFP)^14^, Htt^25Q^ and Htt^46Q^ (Huntingtin)-mCherry (Fig. 1c, *left*), and the M337V mutant of the TAR DNA binding protein-43 fused at the C-terminus with turboGFP (i.e. TDP-43^M337V^-tGFP; Fig. 2b); FloIT was able to quantify inclusions in all cases. The identity of nuclei and inclusions resolved by FloIT was confirmed by collecting these populations using sorting flow cytometry and then examining them by confocal microscopy (Supplementary Fig. S2). A recently described flow cytometric technique estimates the proportion of cells in a population with inclusions by analysing the difference in fluorescence pulse shapes of cells containing diffuse fluorescence versus those with punctate fluorescence associated with large protein inclusions (pulse shape analysis, PulSA)^15^. In our hands, PulSA was able to detect inclusions in transfected cells expressing Htt^46Q^-mCherry and (to a lesser extent) FLUC^DM^-eGFP, but essentially was unable to do so in any of the other models tested, including cells expressing Htt^25Q^-mCherry or SOD1^WT/A4V/G93A^-eGFP (Fig. 1c, *right*; Supplementary Fig. S3). In cells expressing Htt^46Q^ and FLUC^DM^, PulSA detected inclusions in approximately 25% and 8% of cells, respectively, but detected less than 1.5% of cells as containing inclusions when any of the other proteins were expressed. In practice, PulSA is limited to detecting fluorescent inclusions that endow the host cell with, relative to cells lacking inclusions, a reduced fluorescence pulse width and increased fluorescence pulse height (e.g. cells expressing Htt^46Q^-mCherry), but many different inclusion-forming proteins do not fulfil this requirement (Supplementary Fig. S4). In contrast, FloIT liberates the inclusions from cells and then enumerates and characterises the individual fluorescent particles.

**Figure 3:**
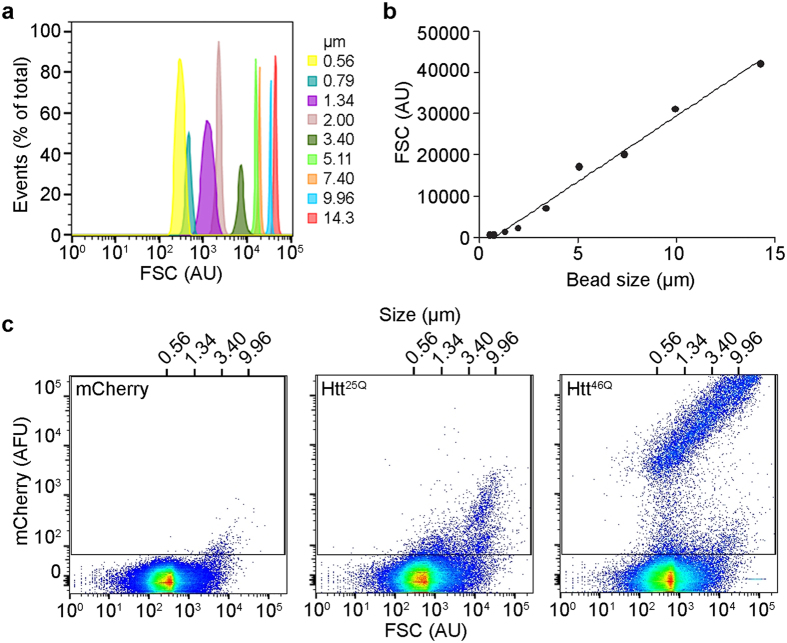
FSC calibration microspheres can be used to estimate the sizes of protein inclusions in FloIT analyses. (**a**) FSC histograms of each of the beads (diameters indicated in key). (**b**) Over the size range tested, FSC and bead size displays a linear relationship (r^2^ = 0.99). (**c**) Pseudocolour plots of analyses of mCherry fluorescence versus FSC for lysates prepared from N2a cells transfected to express mCherry (which does not aggregate to form inclusions, *left*) or Htt^25Q^-mCherry (which forms limited numbers of inclusions, *middle*) or Htt^46Q^-mCherry (which forms large numbers of inclusions, *right*). A range of sizes of inclusions can be resolved, however the absolute sizes of those with diameters less than ~ 0.5 μm cannot be determined using standard flow cytometers (see text). Results shown are representative of three independent experiments.

We confirmed that incubation with 0.5% Triton X-100 rapidly lysed cells but left inclusions intact, and that when lysates were produced at a density of approximately 150,000 cells/ml, samples would return the same FloIT results when analysed up to 3 h post-lysis (Fig. 2). When lysates were produced at twice this cell density (i.e. 300,000 cells/ml), the results of sequential FloIT analyses were stable for a shorter period of time (less than 30 min), which appeared to correspond with settling and aggregation of nuclei and other particles to form macroscopically visible flocculents in the sample (data not shown). To further examine the characteristics of inclusions detected by FloIT, we used a series of microspheres of known diameters to calibrate the FSC signal at the gain setting used for FloIT (Fig. 3a). Over the size range tested (0.56–14.3 μm diameter), the FSC signal showed a linear dependence upon the size of the microspheres, however, it is apparent that the ability to use FSC to resolve between the two smallest beads tested (0.56 and 0.79 μm diameter), is very limited (Fig. 3b). This is expected because, using standard flow cytometry instrumentation, the relationship between FSC and size becomes non-linear for particles of diameter less than the wavelength of the excitation laser (generally 488 nm)^16^. The data presented in Fig. 3c clearly illustrate that FloIT can resolve a broad range of sizes of inclusions, including some less than 500 nm in diameter. However, owing to the technical limitations just discussed, it is not possible to determine the absolute size of these smallest particles using standard instruments.

**Figure 4:**
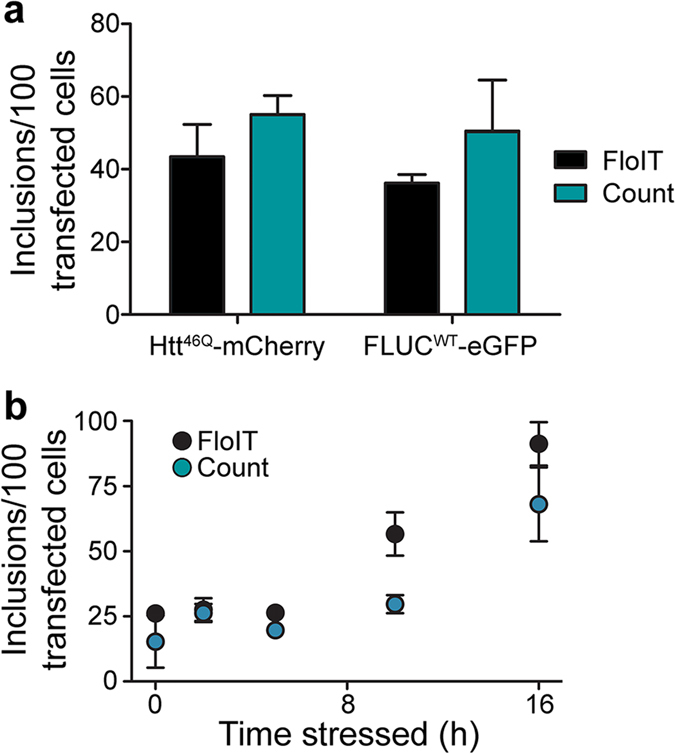
FloIT and manual counting provide similar estimates of the number of inclusions in cells. (**a**) The number of inclusions in N2a cells expressing Htt^46Q^–mCherry or FLUC^WT^–eGFP was enumerated by FloIT or manual counting. Values are means + SEM (n = 3). Differences were not significant, analysed by Student’s t-test. (**b**) Quantification of inclusions in cells expressing TDP-43^M337V^–tGFP, and treated with 10 μM MG132, by FloIT (100,000 events acquired) versus manual counting (3 × 100 cell images). Values plotted are means ± SEM (n = 3). Each result shown is representative of two independent experiments.

**Figure 5:**
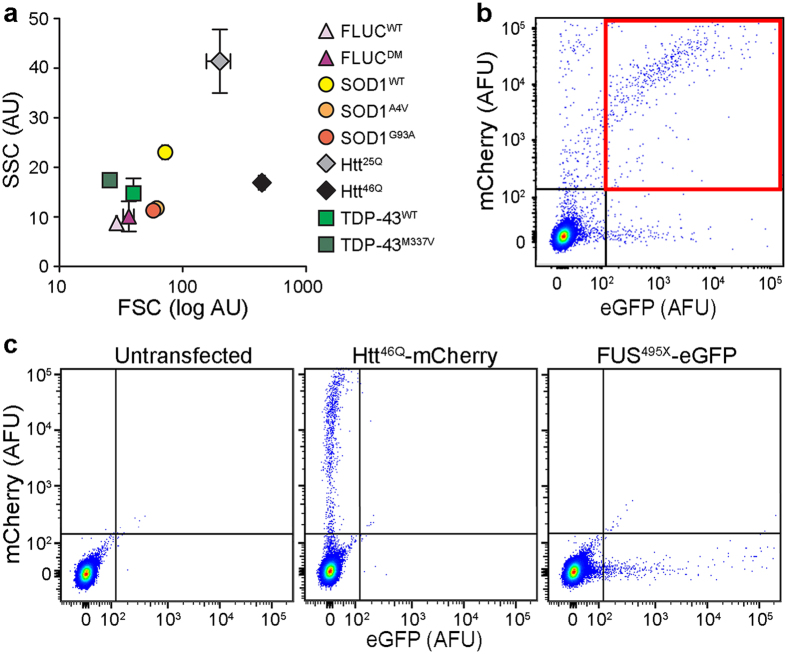
FloIT can resolve different types of protein inclusions. (**a**) FloIT can resolve inclusions formed by different proteins in N2a cells on the basis of FSC and SSC. Values are means (n = 3) ± SEM. (**b**) Inclusions containing two different proteins can be detected by FloIT (in this case FUS^495X^-eGFP and Htt^46Q^-mCherry). Dual-colour particles containing both fusion proteins are within the quadrant indicated by the red square. (**c**) Controls for the data shown in (**b**), including analyses of lysates prepared from untransfected N2a cells (*left*), and N2a cells transfected to express only Htt^46Q^-mCherry (*middle*) or FUS^495X^-eGFP (*right*). Each result shown is representative of two or more independent experiments.

We directly compared FloIT with manual counting of inclusions by epifluorescence microscopy for transfected N2a cells expressing Htt^46Q^-mCherry or FLUC^WT^-eGFP - the numbers obtained using either technique were not significantly different (Fig. 4a). A similar comparison was performed using N2a cells expressing TDP-43^M337V^-tGFP and treated over a 16 h time course with the proteasome inhibitor MG132 to enhance cytoplasmic translocation and aggregation of the fusion protein into inclusions. FloIT detected 26 inclusions/100 transfected cells at t = 0 h, and more than 90 inclusions/100 transfected cells after 16 h of MG132 treatment. At some time points, FloIT indicated more TDP-43-tGFP inclusions in these cells than manual counting (Fig. 4b); factors likely to account for differences of this type in some aggregation models are (i) multiple inclusions that align in the *Z*-axis are not distinguishable when physically counting inclusions from a 2D image (Supplementary Fig. S5), and (ii) FloIT detects very small inclusions that may not be resolved by fluorescence microscopy. As further evidence of the versatility of FloIT, we next showed that on the basis of FSC and side scatter (SSC) signals, it can resolve populations of inclusions formed from many different proteins (Fig. 5a) and, on the basis of fluorescence, resolve and enumerate inclusions formed from different aggregating proteins in the same cells (Fig. 5b; Supplementary Fig. S6). Two additional protein aggregation models are included in these results - the wild type (WT) version of TDP-43-tGFP (i.e. TDP-43^WT^-tGFP, Fig. 5a) and FUS^495X^-eGFP (Fig. 5b).

**Figure 6:**
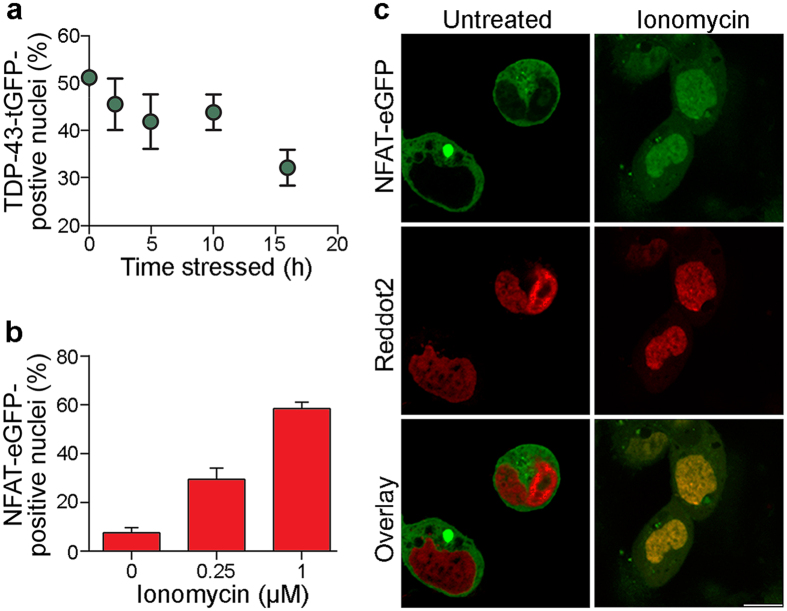
FloIT can quantify nuclear flux of fluorescently tagged proteins. (**a**) Time-dependent efflux of TDP-43^M337V^-tGFP from the nuclei of MG132 treated N2a cells, shown as % change in tGFP-fluorescing nuclei (at t = 0, the % value represents the transfection efficiency). (**b**) Dose-dependent influx of NFAT-eGFP into 1 µM ionomycin-treated HEK293 cell nuclei, shown as % NFAT-eGFP-positive nuclei. (**c**) Confocal microscopy images of NFAT-eGFP-expressing HEK293 cells with and without 1 µM ionomycin treatment. Scale bar is 10 μm. Values are means (n = 3) ± SEM; each result is representative of two or more independent experiments.

We reasoned that FloIT could also be used to measure the trafficking of fluorescently labelled molecules from one cell compartment to another, providing at least one of these compartments remains intact following cell lysis. Two different nuclear trafficking models were used to demonstrate feasibility. Firstly, the efflux of TDP-43^M337V^-tGFP from the nucleus of transfected N2a cells induced by MG132 treatment was measured as a time-dependent decrease in the fluorescence of nuclei. Approximately 51% of nuclei had tGFP fluorescence at t = 0 h and this decreased to approximately 31% at t = 16 h (Fig. 6a). Furthermore, the mean nuclear tGFP fluorescence decreased by approximately 34% over this time period (Supplementary Fig. S7a). Thus, FloIT was able to successfully detect and quantify the MG132-induced efflux of TDP-43^M337V^-tGFP from the nucleus. In a second trafficking model independent of protein aggregation, we used FloIT to measure the movement of a transcription factor (nuclear factor of activated T-cells C-terminally fused to eGFP, i.e. NFAT-eGFP) into the nucleus in response to Ca^2+^ dyshomeostasis induced by ionomycin (a Ca^2+^ ionophore)^17^. In this experiment, transfected HEK293 cells expressing NFAT-eGFP were treated (or not) with ionomycin and subsequently analysed by FloIT. This showed that under the conditions tested 1 μM ionomycin treatment increased (i) the fraction of fluorescent nuclei from 8% to 60% (Fig. 6b), and (ii) the mean fluorescence of nuclei containing NFAT-eGFP by 19-fold (Supplementary Fig. S7b). This movement of NFAT-eGFP into nuclei was confirmed by confocal microscopy (Fig. 6c). Potentially a similar approach could be applied in future to study transport or binding processes involving other cell organelles or structures, providing those organelles can be released intact from cells and are sufficiently large to be resolved by flow cytometry.

In conclusion, FloIT uses standard flow cytometry systems and simple reagents common in most cell biology laboratories to provide a simple yet flexible and powerful method to analyse the formation and physical characteristics of many different protein inclusions (including all those tested relevant to neurodegenerative diseases), and the nuclear trafficking of fluorescent molecules. The ease and power of FloIT offers great potential to: (i) Significantly expand the range and quality of data acquired in the many ongoing studies of proteostasis and neurodegeneration in which protein inclusions are a focus. The ability of FloIT to discriminate between inclusions formed by different proteins, and by one versus more than one protein may find a variety of applications in research. (ii) Provide a platform suitable for medium-throughput drug screening. FloIT could be used, for example, to identify small molecules that inhibit the formation of, or enhance the clearance of, protein inclusions in cells and thereby protect from disease pathologies. (iii) Study transport or binding processes involving cell nuclei, or other cell organelles or structures that can be released intact from cells and are sufficiently large to be resolved by flow cytometry (i.e. larger than around 0.5 μm). By using multiple fluorescent labels, it is envisaged that FloIT could be used to track and quantify dynamic changes in the levels and localisation of multiple molecular species within cells. These additional applications of FloIT are likely to find a very wide range of uses in cell biology, biochemistry and biomedical research.

## Methods

### Tissue Culture

N2a cells were cultured in Dulbecco’s Modified Eagle’s Medium/Ham’s Nutrient Mixture F-12 (DMEM/F12); HEK-293 cells were cultured in high glucose DMEM/F12. Both were supplemented with 10% (v/v) fetal bovine serum (FBS) and cells were incubated at 37 °C and 5% (v/v) CO_2_. Cells were transfected 24 h after plating using Lipofectamine 2000 (Life Technologies) according to the manufacturer’s instructions. Briefly, 1 μg plasmid DNA was used per ~3.5 cm^2^ of cells to be transfected, present at approximately 80% confluency. Transfectants were analysed or exposed to various treatments 24 h (TDP-43 and NFAT experiments) or 48 h (Huntingtin and SOD1 experiments) after transfection.

### Plasmids and cloning

M337V human TDP-43 cDNA was cloned into pCMV6-AC-GFP (Origene) to generate a mutant TDP-43 construct C-terminally tagged with tGFP^18^. HA-NFAT1(1-460)-GFP was a gift from Anjana Rao (Addgene plasmid #11107). Huntingtin protein-encoding constructs, pT-Rex-Htt46Q-Tc1-mCherry and pT-Rex-Htt25Q-Tc1-mCherry, were gifts from Dr. Danny Hatters (University of Melbourne, Australia)^19^. pEGFP-SOD1, pEGFP-SOD1-A4V, pEGFP-SOD1-G93A were gifts from Dr. Brad Turner (The Florey Institute of Neuroscience and Mental Health, Australia)^20^. SOD1-tdTomato constructs were created by replacing the GFP sequences in the SOD1-GFP plasmids with tdTomato (Genscript)^18^. The expression vector pCMV6-AC-GFP containing FUS was obtained from Origene and site directed mutagenesis was performed by Genscript to create the R495X mutant^18^. Plasmids containing sequences encoding wild type (WT) and temperature-sensitive double mutant (R188Q, R261Q; DM) firefly luciferase-eGFP were a gift from Prof Ulrich Hartl (Max Plank Institute, Germany)^21^ and were recloned into pcDNA4/TO (Life Technologies) for mammalian cell transfection.

### FloIT

Cells to be analysed were grown and transfected in 24 well microtitre plates. After the indicated treatments, the cells were harvested using 0.5% (v/v) trypsin/EDTA (Life Technologies), then diluted with either phosphate buffered saline (PBS; 135 mM NaCl, 2.7 mM KCl, 1.75 mM KH_2_PO_4_, pH 7.4) or DMEM/F12 containing 1% FBS and centrifuged (300 × *g* for 5 min at room temperature (RT)), washed once more in PBS and resuspended in 500 μl of PBS. An aliquot of the cell suspension (150 μl) was taken and the transfection efficiency determined by flow cytometric analysis; the fluorescence of transfected cells was compared with that of non-transfected control cells. Flow cytometry was performed using a Becton Dickinson Biosciences LSRFortessa X-20 analytical flow cytometer; excitation wavelengths and emission collection windows were, respectively, e/tGFP (488 nm, 525/50 nm) and mCherry/tdTomato (561 nm, 586/15 nm). The flow cytometer was regularly calibrated using CST beads following the manufacturer’s instructions (Becton Dickinson). The remaining 350 μl of cell suspension was centrifuged as above and resuspended in lysis buffer, comprised of PBS containing 0.5% (v/v) Triton X-100 and Complete protease inhibitor (Roche). Except in control samples used to set gates, RedDot2 (Biotium) was diluted 1:1000 into lysis buffer prior to adding to cells. After a 2 minute incubation at RT to lyse cells, the lysate was immediately analysed by flow cytometry measuring forward and side scatter, together with RedDot2 fluorescence (640 nm excitation, 670/30 nm collection) and e/tGFP and/or mCherry/tdTomato fluorescence as above. The FSC threshold was set to 200 (minimum possible) to minimise exclusion of small inclusions from the analyses. Acquisitions were performed with all axes set to log_10_, and 100,000 events were acquired in all cases. Unless otherwise indicated, voltages of 418 (FSC), 199 (SSC), 410 (e/tGFP), 538 (RedDot2) and 412 (mCherry/tdTomato) were used in all experiments. Nuclei were identified and counted based on RedDot2 fluorescence and FSC. The remaining particles were analysed for the presence of inclusions based on fluorescence, FSC and comparison against an untransfected or vector-only control. The number of inclusions in the population can be normalised to the number of enumerated nuclei, and reported as inclusions/100 transfected cells (*i*) according to equation 1:


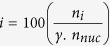


where *n*_*i*_ is the number of inclusions acquired*, n*_*nuc*_ is the number of nuclei acquired, and *γ* is the transfection efficiency. If appropriate, the value for *n*_*nuc*_ in equation 1 can be corrected for any limited nuclear aggregation present (quantified as outlined in Supplementary Fig. S1a). The numbers of nuclear particles identified on the basis of FSC-Area and RedDot2 fluorescence underestimates the actual number of single N2a nuclei by approximately 5%; this correction has been applied to FloIT data presented in this report in units of inclusions/100 nuclei (see Supplementary Fig. S1a, and Results and Discussion text). All analyses of FCS files was performed using FlowJo version 10 (FlowJo).

### PulSA and Manual Inclusion Counting

PulSA was performed as previously described^15^. Briefly, area, height and width parameters were collected for both e/tGFP and mCherry fluorescence using the excitation lasers and bandpass filters outlined above. Plotting fluorescence height against width allows (in some cases) the identification of a population of cells with inclusions in the upper left portion of the cytogram. Manual counting of inclusions was performed by counting the number of inclusions present in three sets of images, each containing 100 randomly selected transfected cells. Images were acquired using a Nikon epifluorescence microscope and a 63x objective.

### Flow Cytometric Sorting of Inclusions and Nuclei

N2a cells grown in 24-well microtitre plates were transfected to express TDP-43^M337V^-tGFP, treated with 10 μM MG132 for 16 h, harvested and lysed as described above. The lysate was passed through a 40 μm nylon mesh and analysed on an S3e Cell Sorter (Bio-Rad Laboratories) equipped with 488 nm and 561 nm lasers. All parameters (fluorescence, FSC and SSC) were acquired on a log_10_ scale. Nuclei were collected based on FSC area and SSC area instead of RedDot2 (since the S3e cytometer used lacked a 640 nm laser); the accuracy of this gate was separately confirmed using propidium iodide (1 μg/ml, excitation 488 nm, emission 586/25 nm). Inclusions were collected based on FSC area and tGFP-fluorescence area (excitation 488 nm, emission 525/30 nm). Particles were collected in 5 ml FACS tubes containing 50 μl of PBS for subsequent imaging using a Leica TCS SP5 II confocal microscope and the Leica Application Suite Advanced Fluorescence software version 2.6.1-7314.

### Nuclear Trafficking Measurements

Transfected N2a cells expressing TDP-43^M337V^-tGFP were treated (or not) with MG132 (10 μM; Cayman Chemicals). After the indicated times, inclusions were measured by either manual counting or FloIT, as described above. In separate experiments, transfected HEK-293 cells expressing NFAT-GFP were treated with ionomycin (Cayman Chemicals) at the indicated concentrations for 30 min. FloIT was performed after this period as described above. For imaging of whole cells, the cells were grown directly in an 8 well μSlide (Ibidi), and following treatment were fixed with 4% (w/v) PFA in PBS for 15 min at room temperature, incubated with RedDot2 and then imaged by confocal microscopy.

## Additional Information

**How to cite this article**: Whiten, D. R. *et al*. Rapid flow cytometric measurement of protein inclusions and nuclear trafficking. *Sci. Rep.* **6**, 31138; doi: 10.1038/srep31138 (2016).

## Supplementary Material

Supplementary Information

Supplementary Movie S1
