# Supplementary material for: Rapid flow cytometric measurement of protein inclusions and nuclear trafficking
Source: Sci Rep. 2016 Aug 12;6:31138. doi: 10.1038/srep31138 (PMC4981889; doi:10.1038/srep31138)
Supplement: Supplementary Information [file srep31138-s1.pdf]

Rapid flow cytometric measurement of protein inclusions and nuclear trafficking

D. R. Whiten[1,2], R. San Gil[1,2], L. McAlary[2], J. J. Yerbury[2], H. Ecroyd[2], M. R. Wilson[2,*]

[1] These authors contributed equally to this work

[2] Illawarra Health and Medical Research Institute and School of Biological Sciences, University of Wollongong, Wollongong NSW 2522

[*] Corresponding author: mrw@uow.edu.au

SUPPLEMENTARY INFORMATION

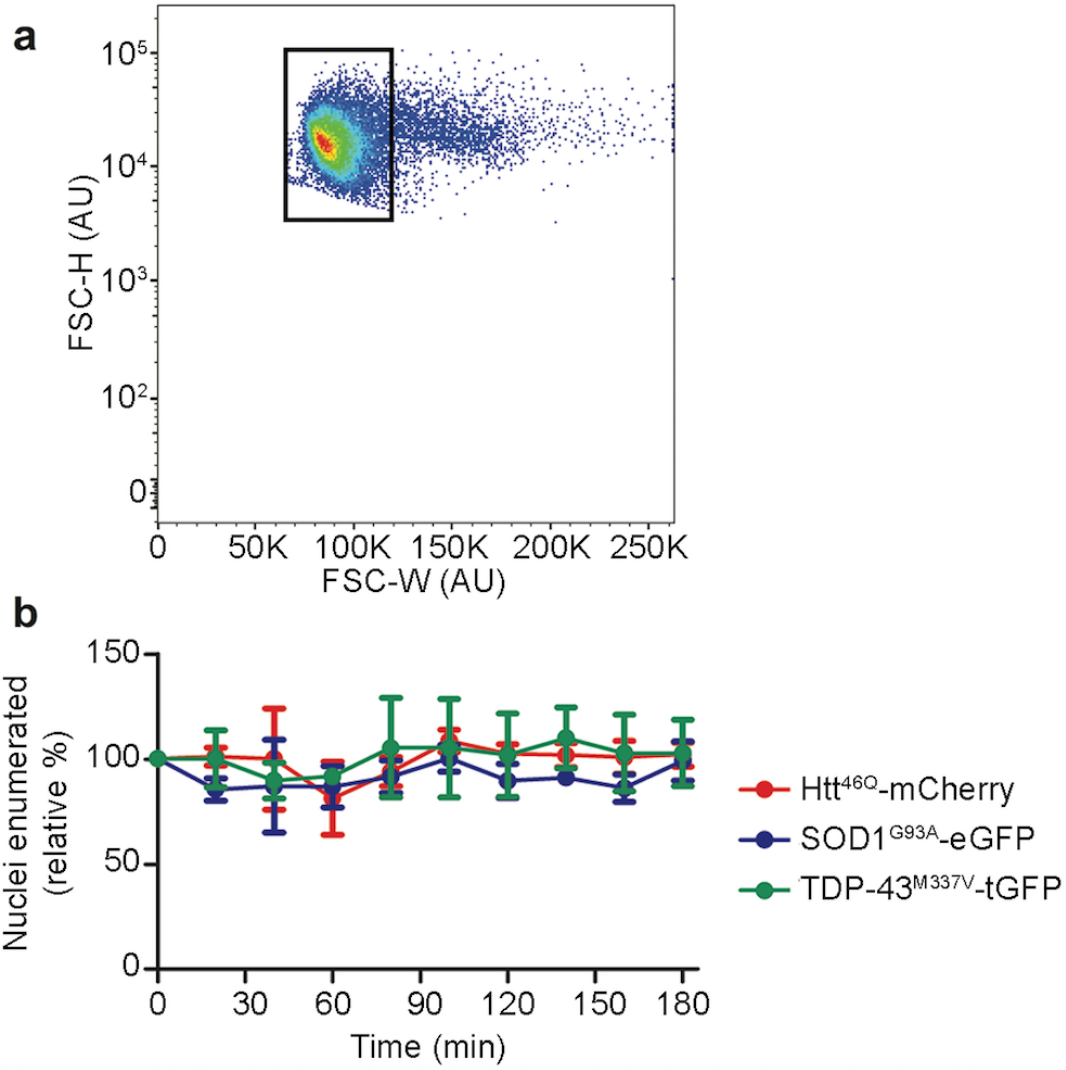

Supplementary Figure S1 Analyses of nuclei in N2a cell lysates. (a) Discrimination of single nuclei versus nuclear doublets. Pseudocolour flow cytometry density plot of FSC-Height (H) versus FSC-Width (W) data for approximately 30,000 nuclear particles in a 30 min old lysate of untransfected N2a cells, prepared as described in Methods (at 150,000 cells/ml), and gated on the basis of RedDot2 fluorescence and FSC-Area (A) (as shown in Fig. 1a, right panel). The particles inside the black rectangle represent single nuclei and in the example shown comprise 94.7% of all events recorded. Nuclear doublets, and small numbers of larger nuclear aggregates, are visible to the right of the black rectangle. The latter comprise only about 0.5% of all RedDot2-stained particles. The result shown is representative of many. **(b)** Under the conditions tested (lysates prepared at 150,000 cells/ml), the number of nuclear particles (gated as in Fig. 1a) enumerated in a 1 min acquisition does not change significantly over a period of several hours. Data is shown for lysates of N2a cells transfected to express one of the three aggregation-prone proteins indicated in the key. Data are plotted as % of t = 0 values and are means ± SEM (n = 3). Results shown are each representative of two independent experiments.

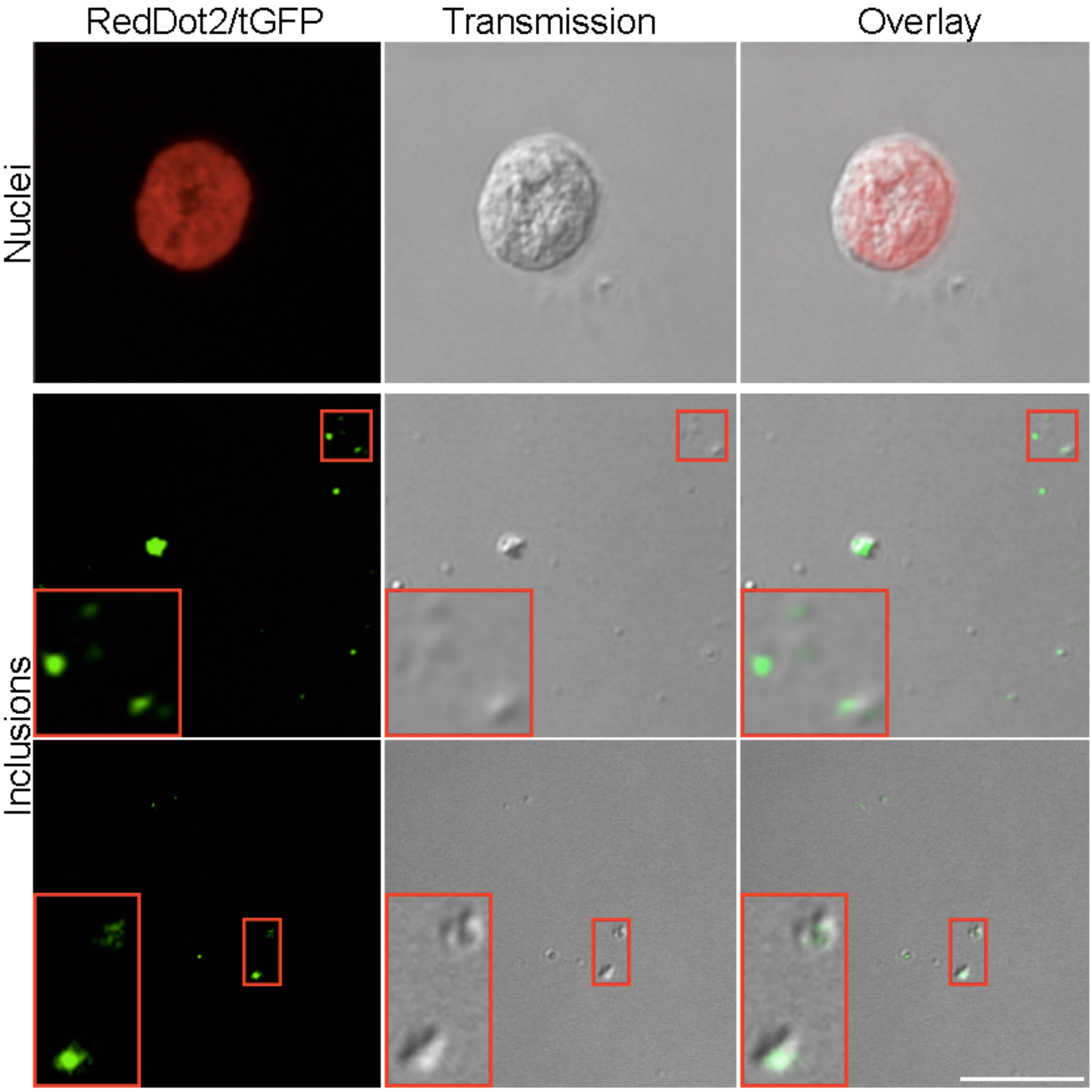

Supplementary Figure S2 Microscopy images of sorted nuclei and inclusions. Transfected N2a cells expressing TDP-43^{M337V}-tGFP were treated for 16 h with 10 µg/ml MG132. Nuclei and TDP-43^{M337V}-tGFP inclusions (identified using FloIT) were collected using a sorting flow cytometer and imaged by confocal microscopy. Scale bar at bottom right is 10 µm. *Lower two rows of panels:* In each image panel, the inset at the bottom left represents a 3X zoom of the area indicated by the small red box to the right. Result shown is representative of two independent experiments.

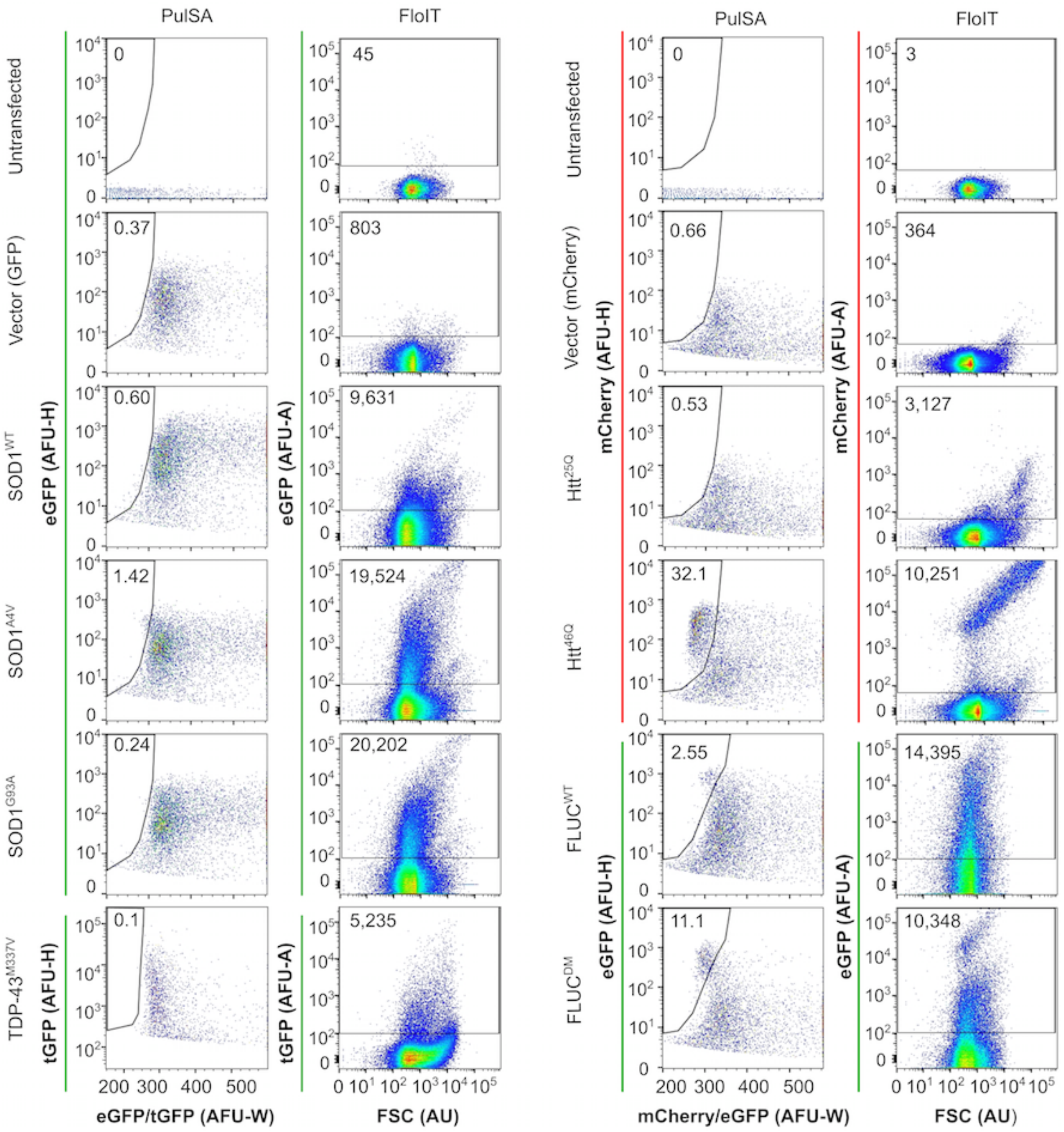

Supplementary Figure S3 Example flow cytometry gates for PulSA (cells with inclusions gated) and FloIT (inclusions from cell lysates gated) used to generate the data presented in Figure 1b. Neuro-2a cells were transfected to express SOD1^{WT}, SOD1^{A4V}, SOD1^{G93A}, TDP-43^{M337V}, Htt^{25Q}, Htt^{46Q}, FLUC^{WT} or FLUC^{DM} fused to the indicated fluorescent protein. Cells transfected with vector alone to express (diffuse) eGFP or mCherry were used as controls to set the PulSA and FloIT gates. Numbers on the PulSA plots denote the percentage of cells resolved as containing inclusions (of 10,000 events acquired), whereas numbers on the FloIT plots denote the actual number of events within the gates (of 100,000 events acquired). *Notes:* The gate drawn for PULSA analysis of TDP-43^{M337V}-tGFP transfected cells (bottom left) is slightly different because of different voltages applied in that analysis. In the FloIT analyses shown in the right hand column, the quadrants drawn to discriminate eGFP-containing inclusions have a slightly higher fluorescence "cut-off" than the quadrants drawn for mCherry-containing inclusions, owing to the slightly higher autofluorescence of lysate particles at the wavelengths collected for eGFP fluorescence. Results shown are representative of two or more independent experiments.

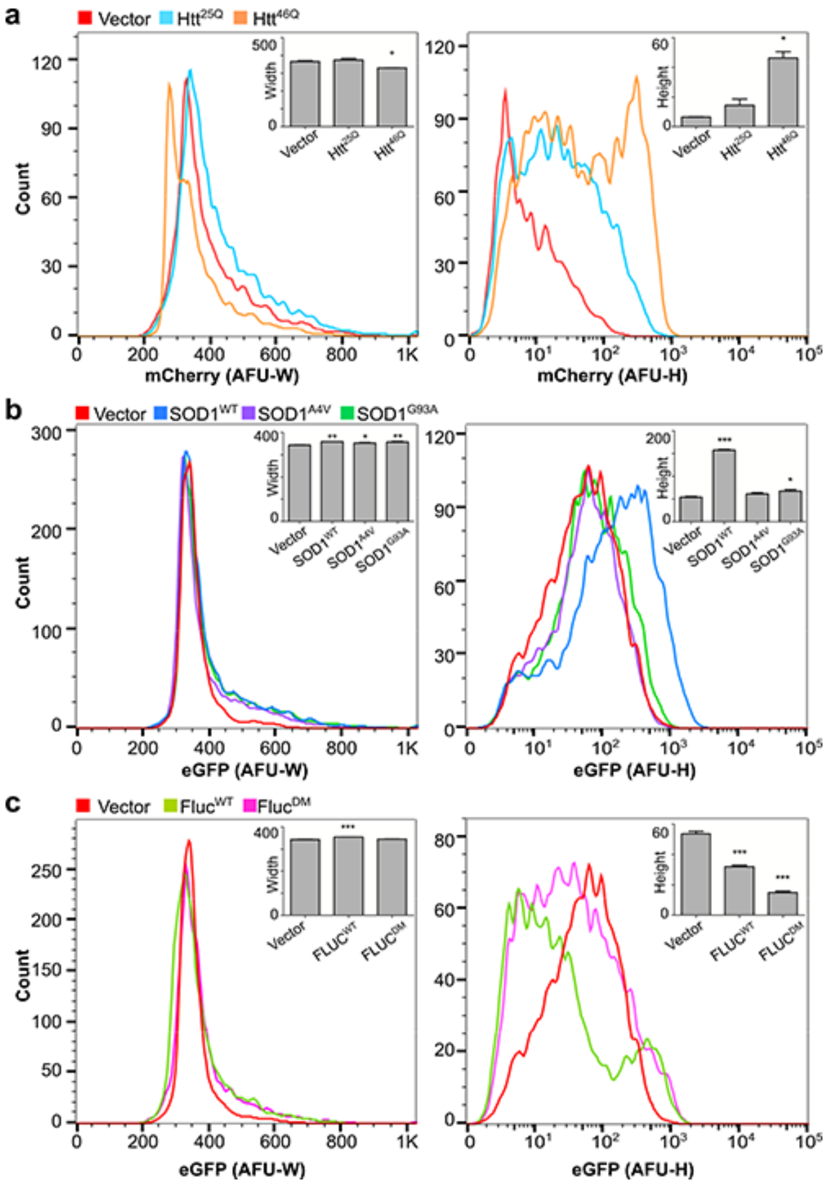

Supplementary Figure S4 Pulse width and height profiles of cells over-expressing a range of aggregation-prone proteins. Overlay frequency histograms of fluorescence pulse width (left panels) and pulse height (right panels) of cells expressing wild-type and aggregation-prone mutants of Htt, SOD1 and FLUC fused to the fluorescent protein indicated on the horizontal axis (10,000 events acquired in all cases). Cells transfected with vector alone to express diffuse eGFP or mCherry were used as controls. **(a)** Cells expressing Htt^{46Q} demonstrated a reduced pulse width and increased pulse height compared to the vector alone control. **(b)** Cells expressing $SOD1^{A4V}$ or $SOD1^{G93A}$ showed an increased pulse width and height compared to the vector control. Lastly, **(c)** relative to the vector control, cells expressing $FLUC^{WT}$ or $FLUC^{DM}$ showed little change in pulse width but showed a decreased pulse height. Therefore not all cells containing protein inclusions conform to the decreased pulse width and increased pulse height paradigm required for identification by PulSA. Insets present a summary of the median fluorescence pulse width and height data. All experiments were performed in N2a cells; values are mean triplicate measurements + SEM. Statistically significant differences between the means were assessed using a one-way ANOVA followed by a Dunnet's post-hoc comparison to vector control, using an alpha level of 0.5 (p<0.05,*; p<0.005, **; p<0.0001,***). Results shown are representative of two or more independent experiments.

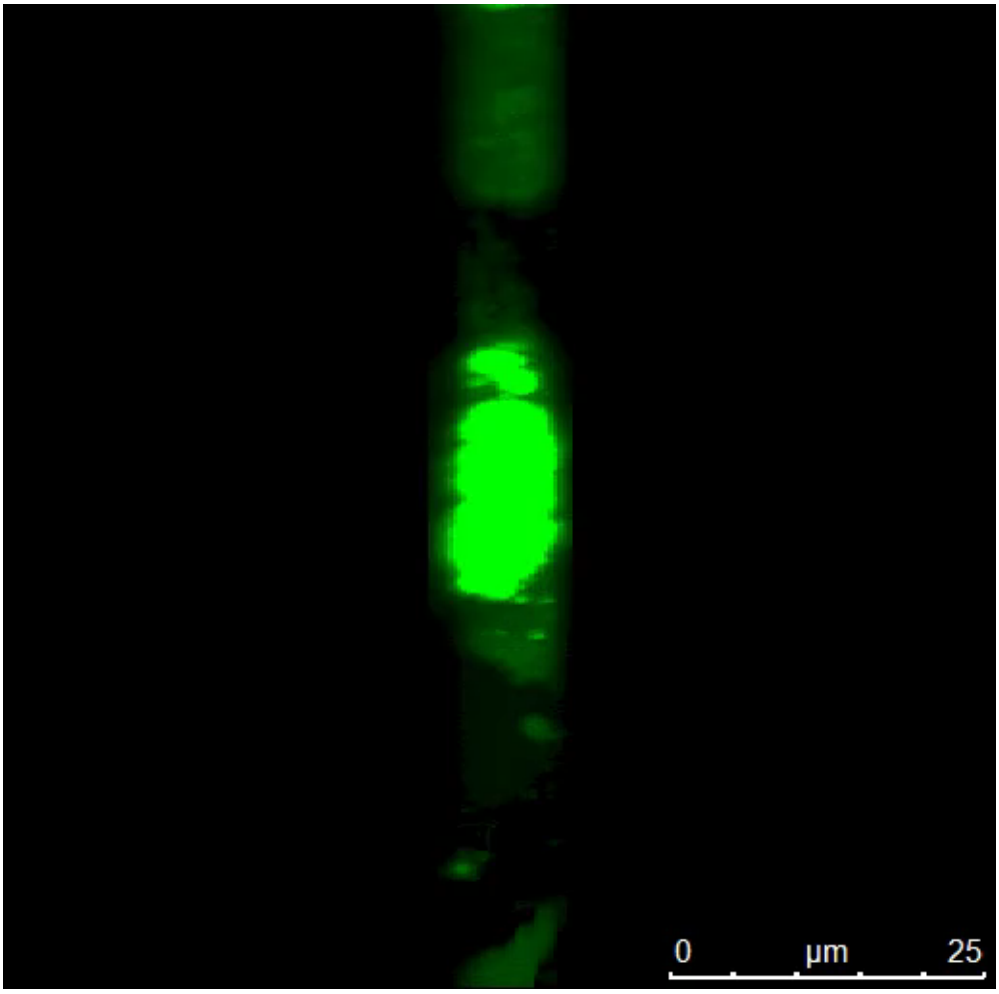

Supplementary Figure S5 In microscopy, the orientation of view can affect the apparent number of inclusions. Video showing rotating three-dimensional image of inclusions within N2a cells transfected to express $SOD1^{G93A}$-eGFP, reconstructed from a "Z-stack" of confocal microscopy images. From some angles of view (as might occur when using epifluorescence microscopy) the cells appear to contain only a small number of large inclusions, however, when the image is rotated the existence of significantly more smaller individual inclusions is apparent. Result shown is representative of three independent experiments. (See separately uploaded QuickTime movie file corresponding to the above image).

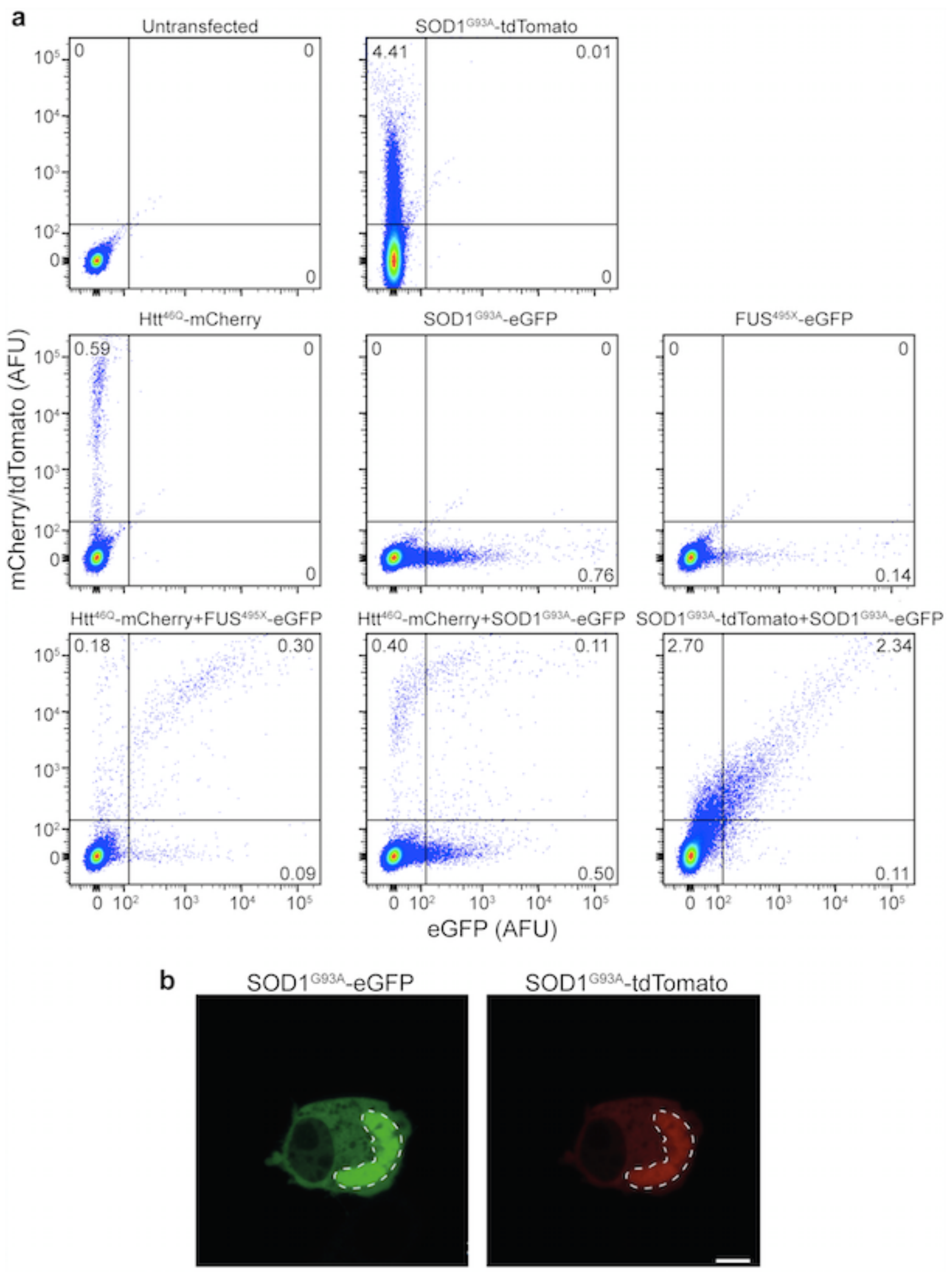

Supplementary Figure S6 FloIT can quantify two-colour inclusions. (a) Flow cytograms of N2a cells overexpressing the protein indicated above each plot and analysed by FloIT. Numbers within the quadrant gates are percentages. **(b)** Example confocal image of an N2a cell exhibiting inclusions containing both SOD1^{G93A}-eGFP and SOD1^{G93A}-tdTomato (indicated by the dotted white line). Scale bar is 5 µm. Result shown is representative of two independent experiments.

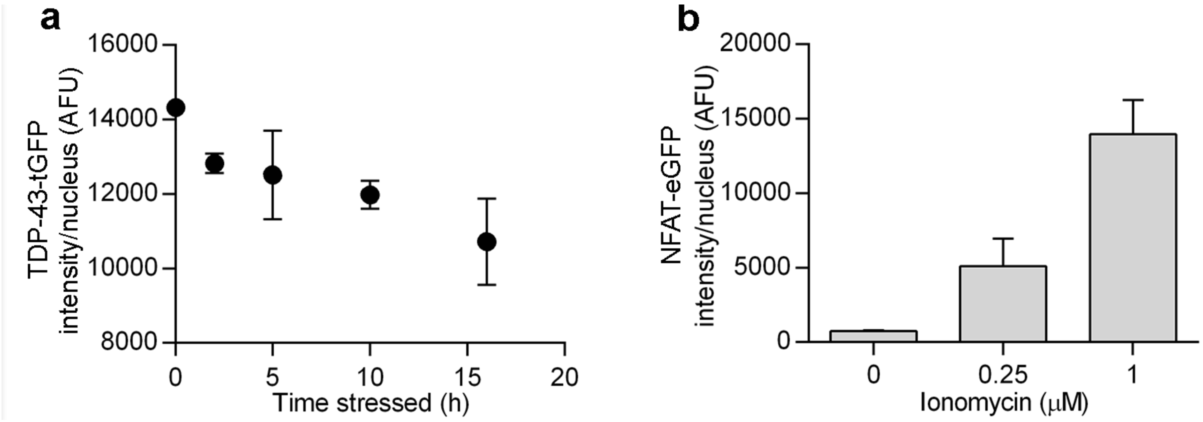

Supplementary Figure S7 FloIT measurements of changes in nuclear fluorescence can be used to quantify the nuclear flux of fluorescently tagged proteins. (a) Time-dependent efflux of TDP-43^{M337V}-tGFP from the nuclei of MG132 treated N2a cells, shown as decreases in the intensity of nuclear TDP-43^{M337V}-tGFP fluorescence. **(b)** Dose-dependent nuclear influx of NFAT-eGFP in ionomycin-treated HEK293 cells, shown as increases in the intensity of nuclear NFAT-eGFP fluorescence. Values are mean (n = 3) ± SEM. Results shown are representative of two or more independent experiments.
